# Meta-Analysis of Pulmonary Artery Denervation for Treatment of Pulmonary Hypertension

**DOI:** 10.21470/1678-9741-2020-0533

**Published:** 2022

**Authors:** Wanyun Zuo, Na Liu, Yunbin Xiao, Yonghui Xie, Qiming Liu

**Affiliations:** 1 Department of Cardiovascular Medicine, The Second Xiangya Hospital of Central South University, Changsha, Hunan, People’s Republic of China; 2 Department of Cardiology, Hunan Children's Hospital, Changsha, Hunan, People’s Republic of China

**Keywords:** Pulmonary Artery, Pulmonary Hypertension, Sympathetic Nervous System, Quality of Life, Heart Ventricles, Meta-Analysis.

## Abstract

**Introduction:**

Pulmonary artery denervation (PADN) can reduce the sympathetic nervous system (SNS) activity, reduce pulmonary artery pressure (PAP), and improve the quality of life in patients with pulmonary hypertension (PH). We conducted a systematic meta-analysis of the effectiveness of PADN in the treatment of PH patients.

**Methods:**

This is a comprehensive literature search including all public clinical trials investigating the effects of PADN on PH. Outcomes were mean pulmonary artery pressure (mPAP), pulmonary vascular resistance (PVR), cardiac output (CO), right ventricular (RV) Tei index, 6-minute walk distance (6MWD), and New York Heart Association (NYHA) cardiac function grading.

**Results:**

A total of eight clinical studies with 213 PH patients who underwent PADN were included. Meta-analysis showed that after PADN, mPAP (mean difference [MD] -12.51, 95% confidence interval [CI] -17.74 to -7.27, *P*<0.00001) (mmHg) and PVR (MD -5.17, 95% CI -7.70 to -2.65, *P*<0.0001) (Wood unit) decreased significantly, CO (MD 0.59, 95% CI 0.32 to 0.86, *P*<0.0001) (L/min) and 6MWD (MD 107.75, 95% CI 65.64 to 149.86, *P*<0.00001) (meter) increased significantly, and RV Tei index (MD -0.05, 95% CI -0.28 to 0.17, *P*=0.63) did not change significantly. Also after PADN, the proportion of NYHA cardiac function grading (risk ratio 0.23, 95% CI 0.14 to 0.37, *P*<0.00001) III and IV decreased significantly.

**Conclusion:**

This meta-analysis supports PADN as a potential new treatment for PH. Further high-quality randomized controlled studies are needed.

**Table:** 

Abbreviations, acronyms & symbols			
6MWD	= 6-minute walk distance	PAP	= Pulmonary artery pressure
CI	= Confidence interval	PH	= Pulmonary hypertension
CO	= Cardiac output	PVR	= Pulmonary vascular resistance
CTEPH	= Chronic thromboembolic pulmonary hypertension	RAAS	= Renin-angiotensin-aldosterone system
EMBASE	= Excerpta Medica dataBASE	RR	= Risk ratios
IL	= Interleukin	RV	= Right ventricular
MD	= Mean differences	RV-LS	= Right ventricular longitudinal peak systolic strain
mPAP	= Mean pulmonary artery pressure	RVFAC	= Right ventricular area change fraction
NOS	= Newcastle-Ottawa Scale	SD	= Standard deviation
NYHA	= New York Heart Association	SE	= Standard error
PADN	= Pulmonary artery denervation	SNS	= Sympathetic nervous system
PAE	= Pulmonary artery endarterectomy	TAPSE	= Tricuspid annular plane systolic excursion
PAH	= Pulmonary arterial hypertension		

## INTRODUCTION

Pulmonary hypertension (PH) is a progressive, extremely malignant, and high-mortality pulmonary vascular disease^[1]^. It is mainly characterized by increased pulmonary vascular resistance (PVR) and continuous increase in pulmonary vascular pressure, which ultimately leads to right heart failure or even sudden death^[2]^. PH can be defined as a rise in pulmonary artery pressure (PAP) induced by various causes, including pre-capillary, post-capillary, and mixed causes^[3]^. The diagnostic criteria for PH is mean PAP (mPAP) ≥ 25 mmHg at rest measured by the right heart catheter at sea level^[3]^. Pulmonary arterial hypertension (PAH), PH caused by left heart disease, PH caused by respiratory disease and/or hypoxia, PH caused by obstructive pulmonary artery disease, and PH caused by unknown factors constitute the current clinical classification of PH^[4]^.

The advent of various new targeted drugs has brought more choices and hopes for the treatment of PH and with the use of targeted drugs, the overall quality of life and survival rate of PAH patients have obviously improved^[5]^. However, most of the current targeted drugs for PAH are vasodilators, and none of them can reverse the progressive pathological remodeling of the pulmonary vessels and right ventricle in PAH patients. In addition, vasodilators did not significantly reduce mortality in the long-term follow-up of PAH patients and some PH patients do not response well to targeted drugs^[6]^. Therefore, it is imperative to actively explore new treatment approach for PH.

A large number of studies have shown that PH is associated with increased sympathetic nervous system (SNS) and renin-angiotensin-aldosterone system (RAAS) activation^[7^,^8]^. SNS originates from the thoracolumbar region of the spinal cord. Short preganglionic fibers from the T1-L2 segments synapse on paravertebral or prevertebral ganglia, enabling long postganglionic fibers to innervate target organs such as the heart and lungs. The activation of SNS and RAAS to produce circulating neurohormone transmitters is an important contributing factor to the progress of PH^[9^,^10]^. Therefore, pulmonary artery denervation (PADN) aimed at reducing SNS activation has become a novel treatment modality^[11^,^12]^. In 2020, a multi-center clinical trial proved that PADN can reduce PVR as well as increase 6-minute walk distance (6MWD) of PAH patients, and no adverse events related to surgery occurred, confirming the effectiveness and safety of PADN^[13]^. However, another clinical study found that the effect of PADN on some PAH patients is not obvious^[14]^. Moreover, the sample size of the current clinical research is too small. Against this background, this meta-analysis aimed to assess the effects of PADN on PH in order to provide evidence-based medical evidence for its clinical application.

## METHODS

This meta-analysis was performed according to recommendations of the Cochrane Handbook for Systematic Reviews of Interventions and Preferred Reporting Items for Systematic Reviews and Meta-Analyses (or PRISMA).

### Search Strategy

Two authors conducted a comprehensive literature search including all human clinical studies of PADN in the treatment of PAH. Literature search was performed with the key words ‘pulmonary artery denervation’ and ‘pulmonary hypertension’ in PubMed and Excerpta Medica dataBASE (or EMBASE).

### Selection Criteria and Exclusion Criteria

Selection criteria included: (1) all randomized clinical trials of which study objects are PH patients; (2) the treatment provided is PADN; (3) the outcomes included (at least one of) mPAP, PVR, cardiac output (CO), right ventricular (RV) Tei index, 6MWD, and New York heart Association (NYHA) cardiac function grading; and (4) there are no restrictions on the language, but valid data can be extracted from the text.

Exclusion criteria included: (1) studies with a sample size of < 10 cases; (2) reviews, animal studies, case reports, and meeting reports; and (3) repeated published literature or periodic report of a research.

### Quality Assessment

Cochrane collaboration’s tool for assessing risk of bias and the Newcastle-Ottawa Scale (NOS) were used to assess the quality of included studies by two independent researchers. The items included in Cochrane collaboration’s tool were random sequence generation, allocation concealment, blinding of participants and personnel, blinding of outcome assessment, incomplete outcome data, and selective reporting and other bias. And the items included in NOS were representativeness of exposed cohort, representativeness of non-exposed cohort, ascertainment of exposure, demonstration that outcome of interest was not present at the start of study, comparability, assessment of outcome, duration of follow-up, and adequacy of follow-up.

### Data Extraction and Outcome Measures

Two independent reviewers performed the data extraction and synthesis. Data extracted from studies included study characteristics, patient characteristics, and outcomes. Outcomes include mPAP, PVR, CO, RV Tei index, NYHA cardiac function grading, and 6MWD.

### Statistical Analysis

This meta-analysis was performed by the Review Manager (RevMan) [Computer program], version 5.3 (Copenhagen: The Nordic Cochrane Centre, The Cochrane Collaboration, 2014). Outcome data were extracted as risk ratios (RRs) and 95% confidence intervals (CIs) or mean differences (MDs) and 95% CIs. Q test and I^^2^^ test were performed to assess the heterogeneity of the included studies^[15]^. When the P-value of Cochran’s Q test was < 0.10 and of I^^2^^ test was > 50%, heterogeneity was considered to exist. Funnel plot was used to evaluate publication bias. Sensitivity analysis was conducted, in which one study was removed at a time to assess the influence of individual studies on results.

## RESULTS

### Literature Search Results

**Fig. 1:**
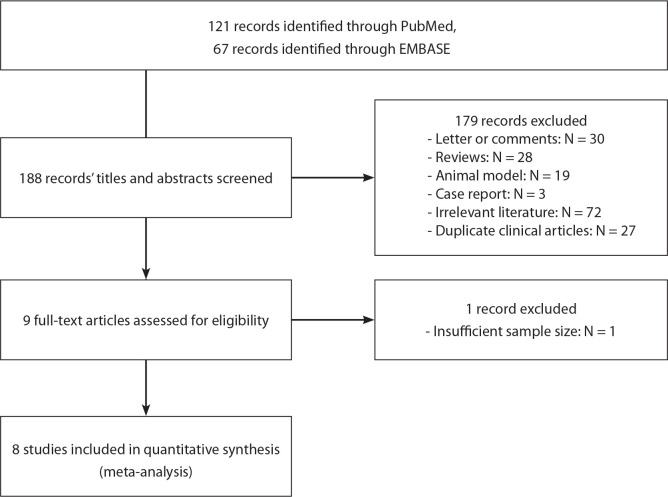
Preferred Reporting Items for Systematic Reviews and Meta-Analyses (or PRISMA) flow chart of study selection. EMBASE=Excerpta Medica dataBASE.

**Fig. 2:**
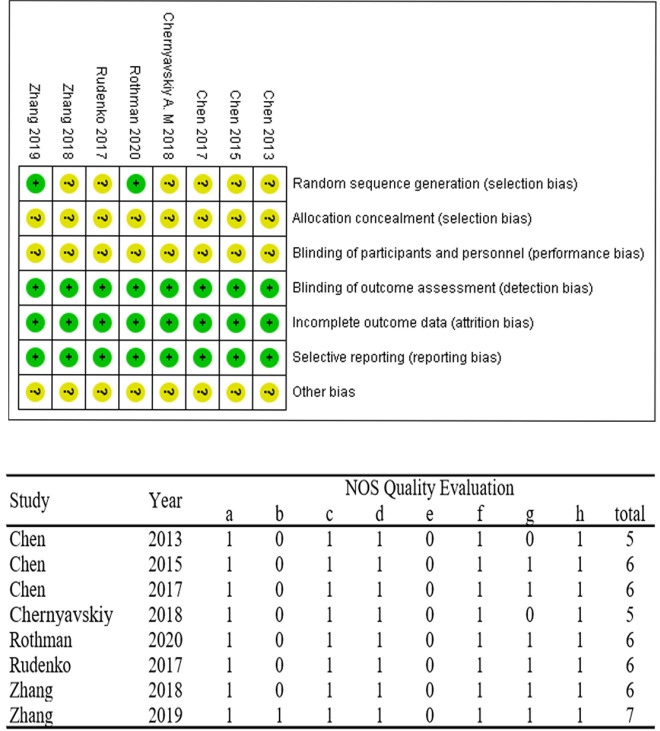
Risk of bias graph: the green color indicates low risk of bias, and the yellow indicates unclear risk of bias. Newcastle-Ottawa Scale (NOS): a=representativeness of exposed cohort; b=representativeness of non-exposed cohort; c=ascertainment of exposure; d=demonstration that outcome of interest was not present at the start of study; e=comparability; f=assessment of outcome; g=duration of follow-up; h=adequacy of follow-up

A total of 188 articles were retrieved and eight studies were finally included, which are all prospective studies and involve a total of 213 patients^[13^,^16^-^22]^. All patients were treated with PADN and followed up for 1-12 months. The literature screening process and quality evaluation are shown in Figure 1 and Figure 2. And characteristics of the included studies are summarized in Table 1.

**Table 1:** Baseline characteristics of included trials.

Study	Year	n	Male/Female	Age	Outcomes
Chen	2013	13	09/abr	40±16	mPAP, PVR, CO, RV Tei index, 6WMD, NYHA
Chen	2015	66	27/39	52±16	mPAP, PVR, CO, RV Tei index, 6WMD
Chen	2017	40	out/30	43±14	mPAP, PVR, 6WMD
Chernyavskiy A. M	2018	16	10/jun	39±18.519	mPAP, PVR, CO, 6WMD
Rothman	2020	23	mai/18	60±11.4	mPAP, PVR, CO, 6WMD
Rudenko	2017	12	06/jun	42±13	mPAP, PVR, 6WMD
Zhang	2018	10	07/mar	67.5±4.7	mPAP, PVR, CO, RV Tei index, 6WMD
Zhang	2019	48	30/18	63.7±11.8	mPAP, PVR, CO, RV Tei index, 6WMD, NYHA

6MWD=6-minute walk distance; CO=cardiac output; mPAP=mean pulmonary artery pressure; NYHA=New York Heart Association; PVR=pulmonary vascular resistance; RV=right ventricular

### Hemodynamic Parameters

**Fig. 3:**
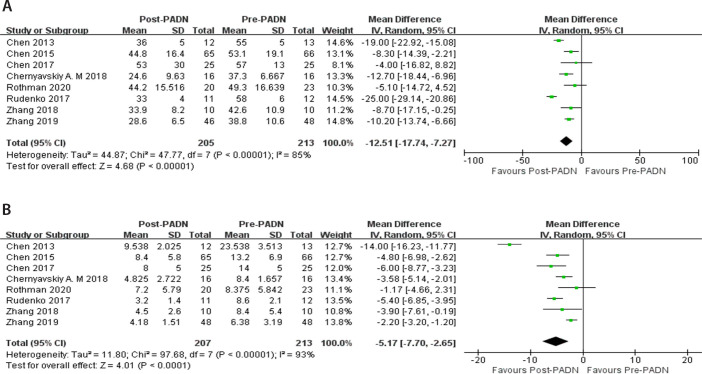
Forest plot for comparison of hemodynamic parameters between post pulmonary artery denervation (PADN) period and pre-PADN period: A) mean pulmonary artery pressure (mmHg); B) pulmonary vascular resistance (Wood unit). CI=confidence interval; SD=standard deviation

Eight studies (n=213) reported the mPAP^[13^,^16^-^22]^. These studies had obvious heterogeneity, so the random effects model was used (*P*<0.00001; I^^2^^ 85%). The results showed that after PADN, mPAP decreased significantly (MD -12.51, 95% CI -17.74 to -7.27, *P*<0.00001) (mmHg) (Figure 3A). Eight studies (n=213) were selected for meta-analysis of PVR ^[13^,^16^-^22]^. Meta-analysis results showed that there was significant heterogeneity between the literatures (*P*<0.00001, I^^2^^ 93%), which was analyzed using a random effects model. The difference between the two groups was significant (MD -5.17, 95% CI -7.70 to -2.65, *P*<0.0001) (Wood unit). Compared with the pre-PADN period, PADN could significantly reduce the PVR of PH patients (Figure 3B).

**Fig. 4:**
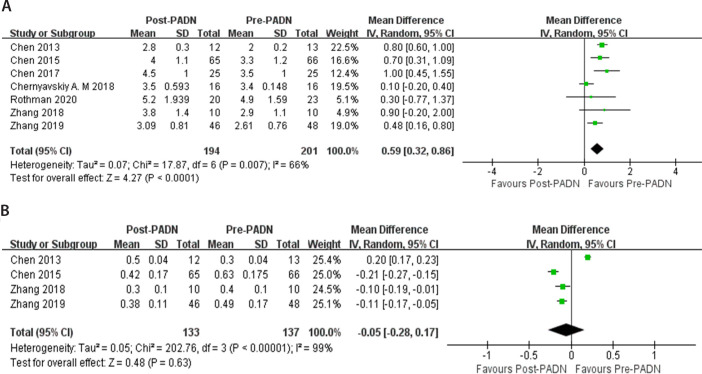
Forest plot for comparison of hemodynamic parameters between post pulmonary artery denervation (PADN) period and pre-PADN period: A) cardiac output (L/min); B) right ventricular Tei index. CI=confidence interval; SD=standard deviation

Seven studies (n=201) reported the CO^[13^,^16^-^19^,^21^,^22]^. These studies had heterogeneity and the random effects model was used (*P*=0.007; I^^2^^ 66%) (L/min). The results indicated that after PADN, CO increased significantly (MD 0.59, 95% CI 0.32 to 0.86, *P*<0.0001) (L/min) (Figure 4A). Four studies (n=137) reported the RV Tei index^[16^,^17^,^21^,^22]^. These studies had heterogeneity and the random effects model was used (*P*<0.00001; I^^2^^ 99%). The results indicated that after PADN, Tei index had no obvious change (MD -0.05, 95% CI -0.28 to 0.17, *P*=0.63) (Figure 4B).

### Quality of Life

**Fig. 5:**
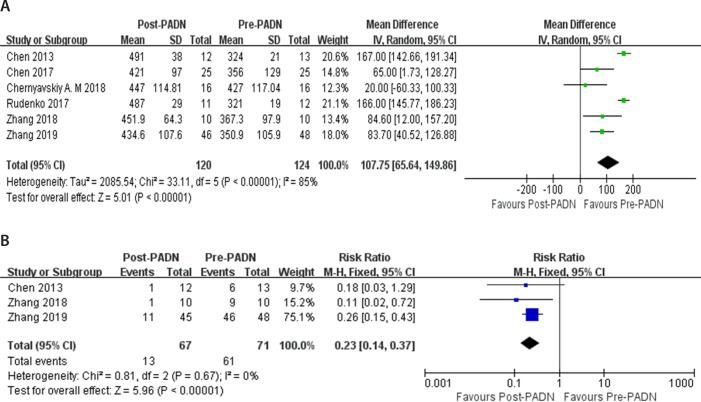
Forest plot for comparison of quality of life between post pulmonary artery denervation (PADN) period and pre-PADN period: A) 6-minute walk distance (meter); B) New York Heart Association cardiac function grading. CI=confidence interval; SD=standard deviation

Five studies (n=124) reported 6MWD^[16^,^17^,^19^-^22]^. There was obvious heterogeneity in these studies (*P*<0.00001; I^^2^^ 85%) and the random effects model was conducted. The results showed that after PADN, 6MWD increased significantly (MD 107.75, 95% CI 65.64 to 149.86, *P*<0.00001) (meter) (Figure 5A). Three studies (n=71) reported changes in NYHA cardiac function grading^[16^,^21^,^22]^. Meta-analysis results showed that the studies were homogenous (*P*=0.67, I^^2^^ 0%), and fixed-effect model analysis was used. There was a significant difference between pre-PADN and post-PADN periods (RR 0.23, 95% CI 0.14 to 0.37, *P*<0.00001). Compared with the pre-PADN period, the proportion of NYHA cardiac function grading III and IV in the post-PADN period decreased (Figure 5B).

### Heterogeneity and Sensitivity Analysis

**Fig. 6:**
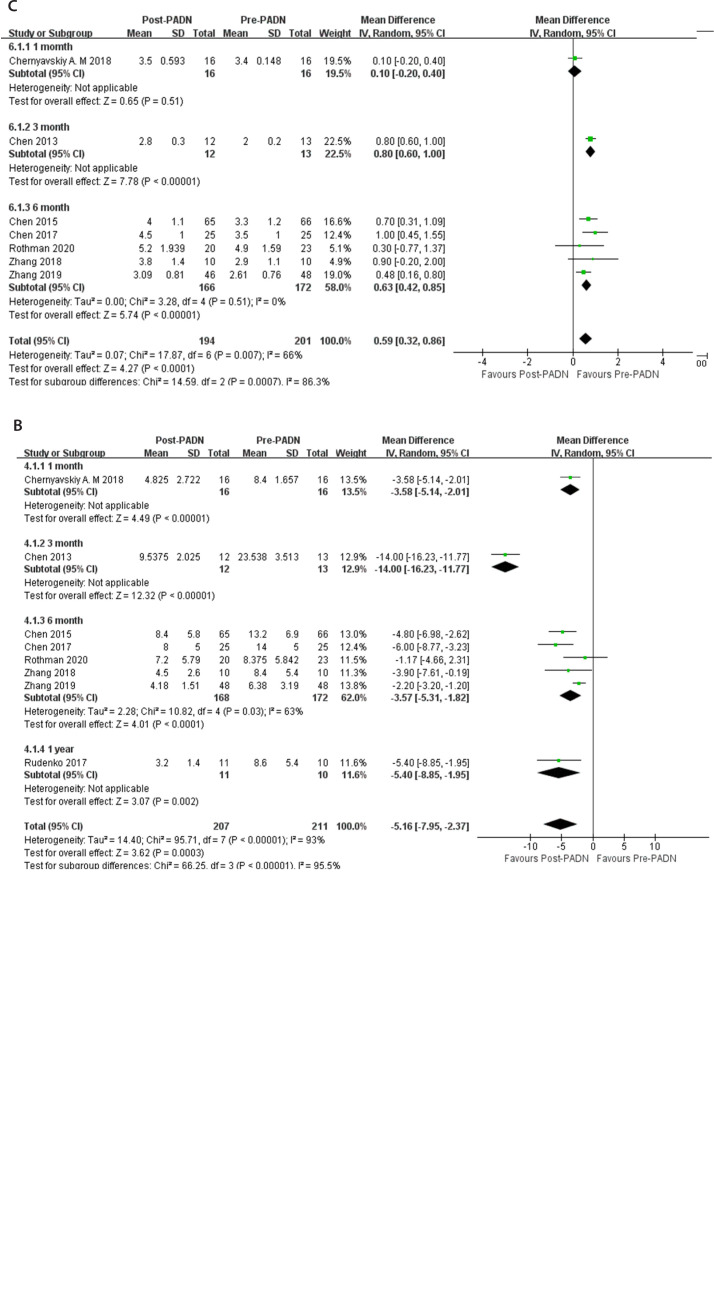
Subgroup analysis: A) mean pulmonary artery pressure (mmHg); B) pulmonary vascular resistance (Wood unit); C) cardiac output (L/min). CI=confidence interval; PADN=pulmonary artery denervation; SD=standard deviation

The results of this meta-analysis showed that the heterogeneity of mPAP, PVR, RV Tei index, and 6MWD was high. After excluding the individual studies one by one, the meta-analysis results showed that the heterogeneity was still high. The difference between pre-PADN and post-PADN periods was statistically significant. This showed that the results of this meta-analysis are relatively reliable. The source of heterogeneity may be related to the different follow-up time and type of PHs between studies. A subgroup analysis of mPAP, PVR, and CO showed that the heterogeneity of five studies with a measurement time of six months was significantly reduced^[13^,^16^,^17^,^21^,^22]^. For mPAP, there was homogeneity (*P*=0.79, I^^2^^ 0%) in five studies with a measurement time of six months, and the difference between the two groups was significant (MD -9.0, 95% CI -11.70 to -6.31, *P*<0.00001) (mmHg) (Figure 6A). For PVR, the heterogeneity of five studies with a measurement time of six months was significantly reduced (*P*=0.03, I^^2^^ 63%). Subgroup analysis showed that compared with the pre-PADN period, PADN could significantly reduce the PVR of PH patients (MD -3.57, 95% CI -5.31 to -1.82, *P*<0.0001) (Wood unit) (Figure 6B). For CO, there was homogeneity (*P*=0.51, I^^2^^ 0%) in five studies with a measurement time of six months, and the difference between the two groups was significant (MD 0.63, 95% CI 0.42 to 0.85, *P*<0.00001) (L/min) (Figure 6C).

### Publication Bias Analysis

**Fig. 7:**
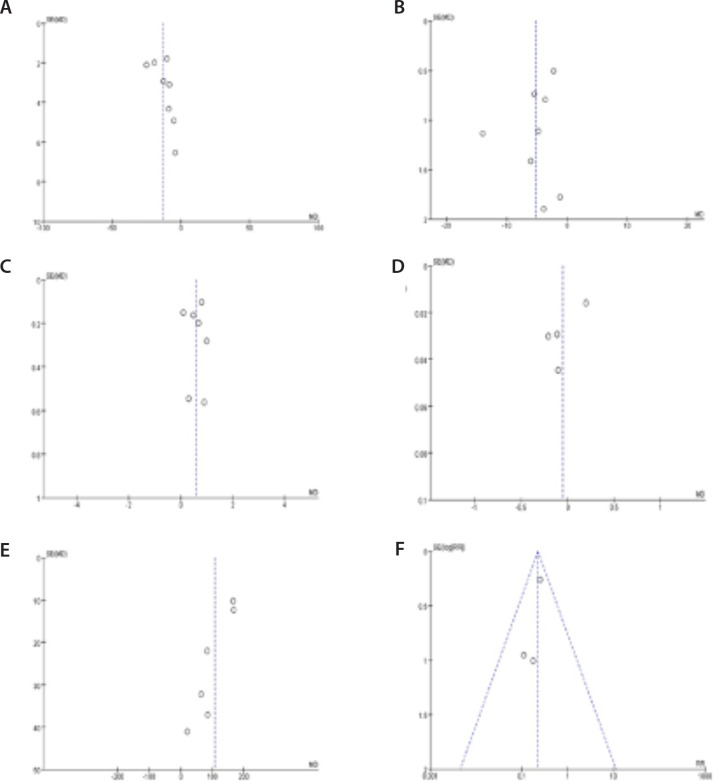
Funnel plots: A) funnel plot of mean pulmonary artery pressure (mmHg); B) funnel plot of pulmonary vascular resistance (Wood unit); C) funnel plot of cardiac output (L/min); D) funnel plot of right ventricular Tei index; E) funnel plot of 6-minute walk distance (meter); F) funnel plot of New York Heart Association cardiac function grading. MD=mean differences; RR=risk ratios; SE=standard error

We used funnel plots for publication bias analysis. The points of the corresponding funnel plots are symmetrical (Figure 7).

## DISCUSSION

To our knowledge, this is the first meta-analysis to evaluate the effect of PADN on PH. We included a total of eight PADN clinical studies with 213 patients with PH. The results showed that after PADN, mPAP and PVR of patients were reduced, CO was significantly increased, but RV Tei index had no obvious changes. 6MWD and cardiac function of PH patients was significantly improved after PADN.

PH is a pulmonary vascular disease with complicated etiology and various treatment methods. There are a number of studies indicating sympathetic excitement involvement in the pathogenesis of PAH models and patients, so PADN targeting SNS could be a therapeutic strategy for PAH and right heart failure. The earliest animal experiment proved that PADN treatment could eliminate PH caused by balloon occlusion of the left interlobar pulmonary artery^[23]^. In porcine and canine PH models, PADN improved the hemodynamics and alleviated RV dysfunction^[24^,^25]^. There were ablation damages to the blood vessels in the ablation zone, including intimal damage, thrombosis, elastic fiber damage, and the reduction of the thickness of middle layer of blood vessel wall in porcine models^[25]^. In the canine model, compared with the sham operation group, the thickness of the vascular wall and the pulmonary muscularization rate decreased in the surgical group, and the pulmonary artery remodeling was significantly improved^[26]^. Besides, PADN could inhibited the messenger ribonucleic acid expression of genes correlated with inflammation, proliferation, and vasoconstriction^[26]^. Huang et al.^[27]^ also proved that serum interleukins (IL)-1β, IL-6, and malondialdehyde levels in the PADN group were significantly lower than those in the sham operation group, and the activity of superoxide dismutase was significantly increased, suggesting that PADN may inhibit lung tissue inflammation and that oxidative stress reduces PAH. The abovementioned animal studies proved that PADN could improve PH hemodynamic parameters, and significantly improved vascular remodeling, reduced RV dysfunction and inflammation, but also caused vascular damage, which provided a basis for the clinical application of PADN. In these animal experiments, for small animals such as rats, researchers often directly remove the SNS around the main pulmonary artery and bifurcations under direct visualization. In large animals, such as dogs, percutaneous catheter intubation for radiofrequency ablation is more commonly used. Obviously, PADN surgery with percutaneous intubation causes less damage and is safer and feasible in humans. In 2013, Chen et al.^[16]^ announced the results of the first percutaneous PADN clinical trial. In this trial, a radiofrequency ablation catheter was inserted into the left pulmonary artery opening of the main pulmonary artery bifurcation through the patient’s femoral vein and was connected to a self-made ablation device, and there were selected three ablation targets, the ostial left pulmonary artery, the distal bifurcation area of the main pulmonary artery, the distal to ostial right pulmonary artery^[16]^. Since then, most clinical studies have adopted percutaneous radiofrequency ablation of PADN. However, the degree of damage to the human pulmonary artery wall and autonomic nerves including parasympathetic nerves due to the ablation energy required during the operation needs to be further studied^[28]^. A multi-center study showed the safety and effectiveness of percutaneous therapeutic intravascular ultrasound for PADN in the treatment of PH^[13]^. The advantages and disadvantages of radiofrequency ablation and ultrasound for PADN require more clinical studies to explore.

This study uses the basic principles and methods of evidence-based medicine to comprehensively analyze the published clinical studies on PH and PADN. This study found that PADN could effectively improve the hemodynamic parameters of PH patients. However, the heterogeneity of these studies is high, and the source of the heterogeneity may be due to the difference in follow-up time, the types of PH, and the use of targeted drugs after PADN of each study.

Some studies have reported that sympathetic nerve regeneration could occur in animal models with PADN, which might be related to sympathetic axon growth mediated by nerve growth factor secreted by abnormally proliferating pulmonary artery smooth muscle cells^[26^,^29]^. Therefore, whether the effect of PADN decays with time deserves further study. Chen et al.^[17]^ showed that all variables of right heart catheterization and 6MWD improved significantly at a six-month follow-up and were non-significantly different between six months and one year. Current clinical studies have been followed up for up to one year, and no effect of PADN has been found to decrease with time^[13^,^17]^. Therefore, we selected the data of six months and the closest follow-up time for six months for meta-analysis. Six-month follow-up studies conducted a subgroup analysis and found that the heterogeneity of mPAP, PVR, and CO was significantly reduced, which indicated that differences in follow-up time might be one of the sources of heterogeneity.

The use of PH-targeted drugs after PADN may also affect outcome indicators, but the studies we included involved both postoperative use and unused PAH targeted drugs. Various studies have shown that regardless of whether PAH targeted drugs are used after surgery, PADN can significantly improve the hemodynamic parameters and the quality of life of PH patients. This meta-analysis also reached the same conclusion, but due to the unclear explanation of the postoperative medication history and insufficient research, no subgroup analysis was performed. As long as a reasonable control group is set up, the use of PAH targeted drugs after surgery will not affect the judgment of the efficacy of PADN.

Studies on PADN improving right heart function and which PH is more suitable for PADN are insufficient. Because RV function plays a critical role in the prognosis of PH patients, measuring RV function is essential to guide treatment and evaluate the progress of the disease^[30^,^31]^. However, there is no accurate index to assess RV function. RV Tei index, tricuspid annular plane systolic excursion (TAPSE), and RV area change fraction (RVFAC) are currently the most commonly used methods for evaluating RV contractile function^[32^-^35]^. This meta-analysis found that PADN did not significantly change the Tei index of PH, which might be due to too little data. Moreover, TAPSE and RVFAC are incomplete, so meta-analysis cannot be performed. In addition, global RV longitudinal peak systolic strain (RV-LS) is another indicator of right heart function, which is closely related to the clinical outcomes of PH patients, and is recommended as the preferred prognostic parameter^[36^-^38]^. Chen et al.^[18]^ reported for the first time the changes of RV function measures after PADN in Group I PAH patients and found that PADN could improve PH hemodynamic parameters, RV functional parameters, and 6MWD, which were related to baseline RV-LS. Specifically, baseline RV-LS ≥ -11.3% might be useful to predict which patients might benefit from PADN^[18]^. More clinical studies are required to assess the benefits of PADN in improving RV function and these parameters that reflect RV function should be valued.

In addition, mechanisms, treatment methods, and responses to treatment of different types of PH are different^[39]^. Apart from targeted drug therapy and etiological treatment, some patients with confirmed chronic thromboembolic pulmonary hypertension (CTEPH) can be cured by pulmonary artery endarterectomy (PAE)^[40^,^41]^. PADN also could be used in in CTEPH patients with residual PH after PAE^[19]^. And the research also proves that PADN has effects on many types of PAH, such as connective tissue disease-related PAH, drug-related PAH, and idiopathic PAH^[13]^. However, due to the lack of current clinical research, most of the types of PH are not separately counted, so we cannot analyze whether there are differential effects of PADN on various PH. Furthermore, PADN could reduce the inflammatory response of PH animal models, but current clinical studies have not compared whether there is a difference in inflammation indicators pre-and post-PADN.

Most studies did not observe the occurrence of surgery-related adverse events, such as pulmonary artery perforation and the formation of dissection aneurysm or acute thrombus^[13^,^16^,^17^,^20]^. Zhang et al.^[22]^ confirmed that compared with the sildenafil group, the improvement of mPAP and 6MWD in the PADN group was more obvious, and the clinical worsening was less frequent. These results confirm the safety and effectiveness of PADN, which can be used to facilitate decision making until the results of larger, controlled studies become available.

### Limitations

There were few limitations in this meta-analysis. Although a large number of studies have demonstrated the potential of PADN to treat PAH, there are few clinical trials to date, so the sample size of this study is relatively small. In addition, there are differences in the types of PH, follow-up time, PADN methods, and whether PAH targeted drugs are used after PADN of these clinical studies. Furthermore, almost all studies included in this meta-analysis are before-after studies in the same patients, which can obtain the difference in the curative effect of the subjects before and after treatment to a certain extent but is more likely to be affected by confounding factors. It is difficult to prove that the difference between before and after treatment is entirely due to the role of surgical intervention. Anyway, in the absence of high-quality randomized controlled studies, the existing evidence of efficacy based on the before-after study in the same patient can still provide a reference for clinical practice.

## CONCLUSION

In conclusion, PADN significantly reduced mPAP and PVR as well as increased CO, but did not increase the Tei index of PH patients. Moreover, PADN increases 6MWD and improved cardiac function of PH patients. In the present meta-analysis, PADN was associated with improved hemodynamics and quality of life of PH patients. Further high-quality randomized controlled studies are needed, and in part ongoing.

**Table:** 

Authors' roles & responsibilities	
WZ	Substantial contributions to the design of the work; and the acquisition, analysis, and interpretation of data for the work; final approval of the version to be published
YX	Substantial contributions to the acquisition of data for the work; final approval of the version to be published
YX	Substantial contributions to the analysis and interpretation of data for the work; final approval of the version to be published
NL	Substantial contributions to the acquisition of data for the work; drafting the work and revising it for important intellectual content; final approval of the version to be published
QL	Substantial contributions to the design of the work; drafting the work and revising it for important intellectual content; final approval of the version to be published
